# Role of the *DLGAP2* Gene Encoding the SAP90/PSD-95-Associated Protein 2 in Schizophrenia

**DOI:** 10.1371/journal.pone.0085373

**Published:** 2014-01-08

**Authors:** Jun-Ming Li, Chao-Lin Lu, Min-Chih Cheng, Sy-Ueng Luu, Shih-Hsin Hsu, Tsung-Ming Hu, Hsin-Yao Tsai, Chia-Hsiang Chen

**Affiliations:** 1 Department of Psychiatry, Taoyuan Armed Forces General Hospital, Taoyuan, Taiwan; 2 Department of Psychiatry, Hualien Armed Forces General Hospital, Hualien, Taiwan; 3 Department of Psychiatry, Yuli Mental Health Research Center, Yuli Branch, Taipei Veterans General Hospital, Hualien, Taiwan; 4 Department of Psychiatry, Chang Gung Memorial Hospital at Linkou and Chang Gung University School of Medicine, Taoyuan, Taiwan; University of Illinois at Chicago, United States of America

## Abstract

Aberrant synaptic dysfunction is implicated in the pathogenesis of schizophrenia. The *DLGAP2* gene encoding the SAP90/PSD-95-associated protein 2 (SAPAP2) located at the post-synaptic density of neuronal cells is involved in the neuronal synaptic function. This study aimed to investigate whether the *DLGAP2* gene is associated with schizophrenia. We resequenced the putative promoter region and all the exons of the *DLGAP2* gene in 523 patients with schizophrenia and 596 non-psychotic controls from Taiwan and conducted a case-control association analysis. We identified 19 known SNPs in this sample. Association analysis of 9 SNPs with minor allele frequency greater than 5% showed no association with schizophrenia. However, we found a haplotype (CCACCAACT) significantly associated with schizophrenia (odds ratio:2.5, p<0.001). We also detected 16 missense mutations and 1 amino acid-insertion mutation in this sample. Bioinformatic analysis showed some of these mutations were damaging or pathological to the protein function, but we did not find increased burden of these mutations in the patient group. Notably, we identified 5 private rare variants in 5 unrelated patients, respectively, including c.−69+9C>T, c.−69+13C>T, c.−69+47C>T, c.−69+55C>T at intron 1 and c.−32A>G at untranslated exon 2 of the *DLGAP2* gene. These rare variants were not detected in 559 control subjects. Further reporter gene assay of these rare variants except c.−69+13C>T showed significantly elevated promoter activity than the wild type, suggesting increased *DLGAP2* gene expression may contribute to the pathogenesis of schizophrenia. Our results indicate that *DLGAP2* is a susceptible gene of schizophrenia.

## Introduction

Schizophrenia is a common and often debilitating mental disorder characterized by hallucination, delusion, bizarre thinking that affects approximately 1% of the general population worldwide. Genetic epidemiological studies from family, twin and adoption studies support a strong genetic component in the etiology of schizophrenia, and the involvement of multiple genes [1], [2], [3]. As far as the pathogenesis of schizophrenia is concerned, increasing evidence demonstrates that aberrant synaptogenesis and synaptic dysfunction play an important role in the pathophysiology of schizophrenia, thus genes involved in the structural and functional integrity of synapse are considered as potential candidate genes of schizophrenia [4], [5], [6], [7].

SAP90/PSD95-associated proteins (SAPAPs) constitute a part of the N-methyl D-aspartate receptor (NMDAR)-associated postsynaptic density proteins, and are involved in the stabilization of synaptic junction and regulation of neurotransmission [8]. The DLGAP gene family, encoding isoforms of SAPAP protein, comprises 4 members, including *DLGAP1*, *2*, *3*, and *4*. Recently, several studies have demonstrated that the DLGAP gene family is involved the pathophysiology of various psychiatric disorders. For example, serial studies supported that the *DLGAP3* gene is a promising functional candidate for Tourette syndrome and obsessive compulsive disorder [9], [10], [11], [12]. Pinto *et al.* discovered the *DLGAP2* gene as a novel gene associated with autism spectrum disorders [13]. Recently, our team has identified a private rare mutation (c.1922A>G) of the *DLGAP1* gene, which changes lysine to arginine at codon 641 (K641R) in one out of 121 schizophrenia, but not in 275 non-psychotic control subjects [14]. In addition, we resequenced the exonic regions of the *DLGAP3* gene in 215 schizophrenic patients and 215 non-psychotic controls. We identified several rare missense mutations in the *DLGAP3* gene and some of them might be associated with the pathogenesis of schizophrenia [15]. Thus, our serial genetic studies of schizophrenia lend support to a hypothesis that schizophrenia specific rare variants in DLGAP gene family may be involved the genetic pathophysiology of schizophrenia.

The *DLGAP2* gene that encodes the SAPAP2 protein also plays a role in the molecular organization of synapses and in neuronal cell signaling [16]. Furthermore, the *DLGAP2* was located on chromosome 8p23, which is a region linked to schizophrenia [17], [18]. This study aimed to examine whether the *DLGAP2* gene is also associated with schizophrenia. To address to this issue, we conducted a deep resequencing study of putative promoter region and all the exons of the *DLGAP2* gene in a sample of schizophrenia and non-psychiatric controls from Taiwan, and characterized the function of variants of the *DLGAP2* gene identified from patients in this study.

## Results

**Figure 1:**
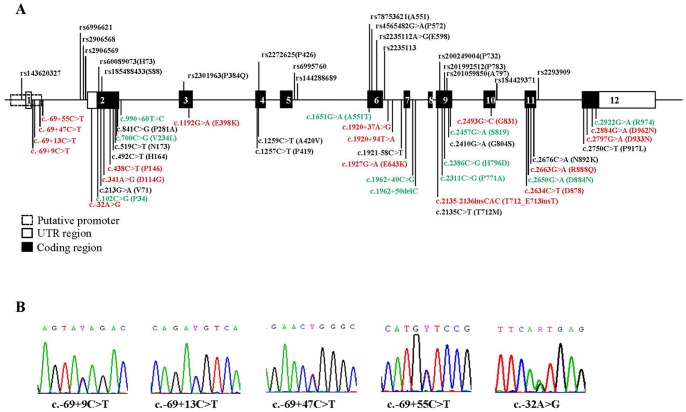
Genetic variants of the *DLGAP2* gene identified in this study. (A) Schematic genomic structure of the *DLGAP2* gene and locations of molecular variants analyzed in this study. The black box indicates the protein-coding region; the white box indicates the untranslated region. Variants represented in red were specific to schizophrenia and in green were specific to the controls. (B) Sequence electropherograms of five private rare variants (c.−69+9C>T, c.−69+13C>T, c.−69+47C>T, c.−69+55C>T and c.−32A>G) at the intron 1 and untranslated exon 2 of the *DLGAP2* gene.

**Figure 2:**
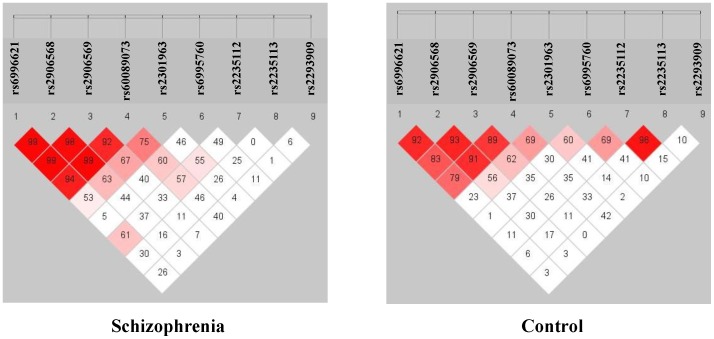
Plots of pair-wise linkage disequilibrium of the 9 SNPs of the *DLGAP2* gene in the schizophrenic patients and controls.

We totally identified 58 genetic variants of the *DLGAP2* gene in this sample, including 19 known SNPs and 39 rare mutations (minor allele frequency <5%). The locations of these variants are shown in Figure 1A. Nine known SNPs (rs6996621, rs2906568, rs2906569, rs60089073, rs2301963, rs6995760, rs2235112, rs2235113, and rs2293909) with the minor allele frequencies (MAF) above 5% were selected for genetic association analysis. The genotype and allele frequencies of these 9 SNPs are listed in Table 1. A nominal association of 4 SNPs (rs2906568, rs2906569, rs2301963 and rs2293909) with schizophrenia was detected; however, this association did not endure after correction for multiple testing. Linkage disequilibrium (LD) analysis of these 9 SNPs showed strong LD among rs6996621, rs2906568, rs2906569, and rs60089073 (Figure 2). Further haplotype-based association analysis derived from these 9 SNPs showed a significant difference in the haplotype distribution of CCACCAACC, CCACCAACT, and CGGCAAACT between the schizophrenic patients and controls, but only haplotype CCACCAACT remained significantly associated with schizophrenia after Monte Carlo permutation test (odds ratio = 2.5, p<0.001) (Table 2).

**Table 1:** Genotype and allele frequencies of molecular variants of the *DLGAP2* gene in patients with schizophrenia and controls.

Variant	Diagnosis	N	Genotype			p	Allele		p
rs6996621			C/C	C/A	A/A	0.327	C	A	0.747
c.−68−61C>A	Schizophrenia	503	421(83.7%)	78(15.5%)	4(0.8%)		920(91.5%)	86(8.5%)	
	Control	559	469(83.9%)	80(14.3%)	10(1.8%)		1018(91.1%)	100(8.9%)	
rs2906568			C/C	C/G	G/G	0.043	C	G	0.109
c.−68−25C>G	Schizophrenia	506	183(36.2%)	239(47.2%)	84(16.6%)		605(59.8%)	407(40.2%)	
	Control	559	197(35.2%)	236(42.2%)	126(22.5%)		630(56.4%)	488(43.6%)	
rs2906569			A/A	A/G	G/G	0.024	A	G	0.025
c.−68−4A>G	Schizophrenia	506	211(41.7%)	227(44.9%)	68(13.4%)		649(64.1%)	363(35.9%)	
	Control	559	215(38.5%)	234(41.9%)	110(19.7%)		664(59.4%)	454(40.6%)	
rs60089073			C/C	C/T	T/T	0.479	C	T	0.698
p.H53 =	Schizophrenia	506	437(86.4%)	62(12.3%)	7(1.4%)		936(92.5%)	76(7.5%)	
	Control	559	483(86.4%)	63(11.3%)	13(2.3%)		1029(92.0%)	89(8.0%)	
rs2301963			C/C	C/A	A/A	0.042	C	A	0.038
p.P384Q	Schizophrenia	510	154(30.2%)	259(50.8%)	97(19.0%)		567(55.6%)	453(44.4%)	
	Control	596	140(23.5%)	330(55.4%)	126(21.1%)		610(51.2%)	582(48.8%)	
rs6995760			A/A	A/G	G/G	0.381	A	G	0.703
c.1570+14A>G	Schizophrenia	508	354(69.7%)	131(25.8%)	23(4.5%)		839(82.6%)	177(17.4%)	
	Control	533	370(69.4%)	147(27.6%)	16(3.0%)		887(83.2%)	179(16.8%)	
rs2235112			A/A	A/G	G/G	0.517	A	G	0.271
p.E598 =	Schizophrenia	523	74(14.1%)	255(48.8%)	194(37.1%)		403(38.5%)	643(61.5%)	
	Control	514	66(12.8%)	240(46.7%)	208(40.5%)		372(36.2%)	656(63.8%)	
rs2235113			C/C	C/G	G/G	0.917	C	G	0.789
c.1920+19C>G	Schizophrenia	512	127(24.8%)	250(48.8%)	135(26.4%)		504(49.2%)	520(50.8%)	
	Control	510	121(23.7%)	254(49.8%)	135(26.5%)		496(48.6%)	524(51.4%)	
rs2293909			T/T	T/C	C/C	0.057	T	C	0.021
c.2709+71T>C	Schizophrenia	507	264(52.1%)	211(41.6%)	32(6.3%)		739(72.9%)	275(27.1%)	
	Control	575	265(46.1%)	256(44.5%)	54(9.4%)		786(68.3%)	364(31.7%)	

**Table 2:** The distributions of *DLGAP2* haplotypes in schizophrenia and controls.

	Haplotype	Cases (freq)	Controls (freq)	Chi2	Pearson’s p	Monte Carlo p	Odds ratio [95% CI]
1	ACACAAGGT	21.78(0.0329)	27.05(0.0354)	0.1462	0.7022	1.0000	0.8957 [0.5090,1.5760]
2	CCACCAACC	31.78(0.0480)	66.01(0.0864)	9.8839	0.0017	0.3000	0.5045 [0.3273,0.7777]
3	CCACCAACT	111.94(0.1691)	58.83(0.0770)	29.4765	0.0000	<0.0010	2.5000 [1.7840,3.5035]
4	CCACCAGGC	24.76(0.0374)	26.74(0.0350)	0.0179	0.8936	1.0000	1.0381 [0.5998,1.7967]
5	CCACCAGGT	70.90(0.1071)	85.03(0.1113)	0.2303	0.6313	1.0000	0.9211 [0.6583,1.2887]
6	CCATCAACT	36.71(0.0555)	26.89(0.0352)	3.1951	0.0739	1.0000	1.5758 [0.9537,2.6035]
7	CGGCAAACT	14.23(0.0215)	31.17(0.0408)	5.0436	0.0247	1.0000	0.4955 [0.2655,0.9247]
8	CGGCAAGGC	26.35(0.0398)	45.61(0.0597)	3.6629	0.0556	1.0000	0.6247 [0.3845,1.0150]
9	CGGCAAGGT	72.49(0.1090)	82.36(0.1078)	0.0185	0.8917	1.0000	0.9769 [0.6977,1.3678]
10	CGGCAGGCT	20.52(0.0310)	37.44(0.0490)	3.6132	0.0573	1.0000	0.5950 [0.3466,1.0215]
11	CGGCAGGGT	23.63(0.0357)	21.93(0.0287)	0.4371	0.5086	1.0000	1.2162 [0.6802,2.1744]

Global result: Total control = 662, total case = 764; Global chi^2^ is 48.4847 while df = 10 (frequency <0.03 in both control & case has been dropped.); Fisher’s p value is 0.0000; Pearson’s p value is 0.0000; Permutation p value (Fisher) is 0.0000; Permutation p value (Pearson) is 0.0000; 1000 permutations; Website: http://analysis.bio-x.cn/myAnalysis.php.

Thirty nine rare variants include 16 missense variants (p.D114G, p.V234L, p.P281A, p.E398K, p.A421V, p.E643K, p.T712M, p.P771A, p.H796D, p.G804S, p.D884N, p.R888Q, p.N892K, p.P917L, p.D933N, and p.D962N) and 1 amino acid insertion mutation (p.T712_E713insT) and are listed at Table 3. Among these 17 rare variants, 7 (p.D114G, p.E398K, p.E643K, p.T712_E713insT, p.R888Q, p.D933N and p.D962N) were detected in schizophrenic patients only, while 5 (p.V234L, p.A421V, p.P771A, p.H796D, and p.D884N) were detected in control subjects only. Five missense mutations (p.P281A, p.T712M, p.G804S, p.N892K, and p.P917L) were detected in both patients and control subjects. There is no increased burden of these rare mutations in the patient group as compared to the control group (16/523 cases versus 16/596 controls, p = 0.715). The functional impact of these missense mutations was assessed by computer programs Polyphen-2 and Pmut. Six missense mutations (p.D114G, p.281A, p.H796D, p.G804S, p.N892K, and p.D962N) were predicted to be both damaging (possibly or probably) and pathological using PolyPhen-2 and Pmut softwares. Two missense mutations (p.P281A and p.D933N) were predicted to be probably damaging to protein function using PolyPhen-2, while 4 (p.P771A, p.D884N, p.R888Q, and p.917L) were predicted to be pathological effect using Pmut (Table 3).

**Table 3:** Distributions and bioinformatic analyses of rare variants of the *DLGAP2* gene identified in this study.

Variants	Frequencies		Amino acidchange	*In silico* analysis		
	Schizophrenia	Control		Transcription factor^a^	PolyPhen-2 (score)^b^	Pmut (NN output)^c^
c.−69+9C>T	1/511	0/559	Intron	None	Na	Na
c.−69+13C>T	1/511	0/559	Intron	ATF, HBP, CREB	Na	Na
c.−69+47C>T	1/511	0/559	Intron	None	Na	Na
c.−69+55C>T	1/511	0/559	Intron	None	Na	Na
c.−32A>G	1/506	0/559	5′UTR	c-Myb, EcR	Na	Na
c.102C>G	0/506	1/559	p.P34 =	Na	Na	Na
c.213G>A	1/506	1/559	p.V71 =	Na	Na	Na
c.341A>G	1/506	0/559	p.D114G	Na	Probably damaging (1.000)	Pathological (0.8216)
c.438C>T	1/505	0/559	p.P146 =	Na	Na	Na
c.492C>T	3/511	8/564	p.H164 =	Na	Na	Na
c.519C>T	1/511	3/564	p.N173 =	Na	Na	Na
c.700G>T	0/511	2/564	p.V234L	Na	Benign (0.046)	Neutral (0.2941)
c.841C>G	2/511	5/564	p.P281A	Na	Probably damaging (0.996)	Neutral (0.2332)
c.990+60T>C	1/204	0/562	Intron	None	Na	Na
c.1192G>A	2/510	0/596	p.E398K	Na	Benign (0.017)	Neutral (0.4983)
c.1257C>T	0/500	1/547	p.P419 =	Na	Na	Na
c.1262C>T	0/500	1/547	p.A421V	Na	Benign (0.000)	Neutral (0.4405)
c.1650G>A	0/523	1/515	p.T551 =	Na	Na	Na
c.1920+37A>G	2/512	0/507	Intron	None	Na	Na
c.1920+94T>A	1/500	0/502	Intron	None	Na	Na
c.1921−58C>T	2/505	1/565	Intron	None	Na	Na
c.1927G>A	1/505	0/565	p.E643K	Na	Benign (0.028)	Neutral (0.2677)
c.1962+40C>G	0/505	1/565	Intron	None	Na	Na
c.1962+50delC	0/505	2/565	Intron	None	Na	Na
c.2135C>T	1/502	2/553	p.T712M	Na	Benign (0.011)	Neutral (0.4149)
c.2135−2136 insCAC	1/502	0/553	p.T712_E713insT	Na	Na	Na
c.2311C>G	0/502	1/553	p.P771A	Na	Benign (0.282)	Pathological (0.5402)
c.2386C>G	0/502	1/553	p.H796D	Na	Probably damaging (0.999)	Pathological (0.8971)
c.2410G>A	1/502	1/553	p.G804S	Na	Probably damaging (0.881)	Pathological (0.8112)
c.2457 G>A	0/502	1/553	p.S819 =	Na	Na	Na
c.2493G>C	1/513	0/544	p.G831 =	Na	Na	Na
c.2634C>T	1/507	0/575	p.D878 =	Na	Na	Na
c.2650G>A	0/507	1/575	p.D884N	Na	Benign (0.222)	Pathological (0.7218)
c.2663G>A	1/507	0/575	p.R888Q	Na	Benign (0.003)	Pathological (0.6063)
c.2676C>A	1/507	1/575	p.N892K	Na	Possibly damaging (0.367)	Pathological (0.8349)
c.2750C>T	3/500	1/549	p.P917L	Na	Benign (0.002)	Pathological (0.0120)
c.2797G>A	1/500	0/549	p.D933N	Na	Probably damaging (0.999)	Neutral (0.3196)
c.2884G>A	1/500	0/549	p.D962N	Na	Probably damaging (0.999)	Pathological (0.6800)
c.2922G>A	0/500	2/549	p.R974 =	Na	Na	Na

^a^ TESS (http://www.cbil.upenn.edu/cgi-bin/tess/tess).

^b^ PolyPhen-2 (http://genetics.bwh.harvard.edu/pph2/).

^c^ Pmut (http://mmb2.pcb.ub.es:8080/PMut/).

Na = not available.

Furthermore, we identified 4 private rare variants (c.−69+9C>T, c.−69+13C>T, c.−69+47C>T, c.−69+55C>T) at the intron 1 and 1 private variant (c.−32A>G) at the untranslated exon 2 of the *DLGAP2* gene in 5 respective patients. These variants were not found in 559 control subjects. The sequencing results are shown in Figure 1B. Bioinformatic analysis predicts that the c.−69+13C>T may change transcription factor binding sites of ATF, HBP, CREB, and the c.−32A>G may change transcription factor binding sites of c-Myc, EcR (Table 3).

**Figure 3:**
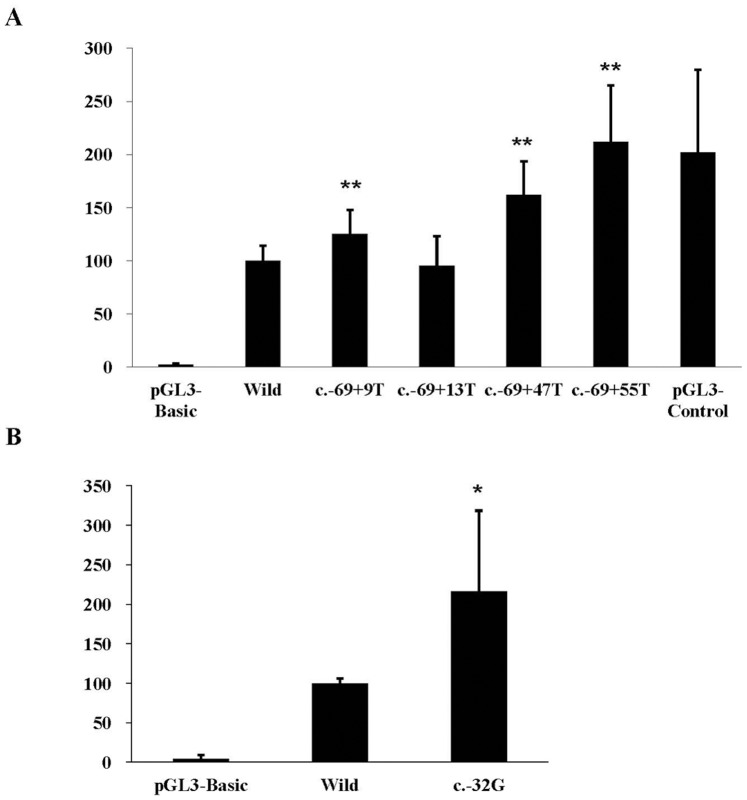
Reporter gene assay of the identified rare variants at the intron 1 and untranslated exon 2 of the *DLGAP2* gene. (A) The promoter activity of three variants c.−69+9T, c.−69+47T, c.−69+55T had significantly higher than that wild type of the *DLGAP*2 gene in SKNSH cells. (B) The promoter activity of allele c.−32G is significantly higher than that wild type of the *DLGAP2* gene in SKNSH cells. **p<0.01; *p<0.05.

We further performed a reporter gene activity assay to assess the potential regulatory impact of these 5 variants (c.−69+9C>T, c.−69+13C>T, c.−69+47C>T, c.−69+55C>T and c.−32A>G) on the expression of the *DLGAP2* gene. As shown in Figure 3, 4 variants (c.−69+9T, c.−69+47T, c.−69+55T and c.−32G) showed significantly increased promoter activity compared to the wild type in SKNSH cells, but this elevation of reporter gene activity was not observed in the c.−69+13T variant.

## Discussion

Schizophrenia is a complex genetic disorder with the involvement of multiple genes. The common variants hypothesis proposes that schizophrenia is attributed to the combined effect of multiple common variants, each with a small-to modest disease risk [19]. Alternatively, the rare mutation hypothesis of schizophrenia suggests that schizophrenia is attributed to rare mutations with large effect in multiple genes in different patients [20]. These two hypotheses are not necessarily mutually exclusive, rare mutations with large clinical effect may occur in genes that also harbor common variants with small disease risk [19], [20]. In this study, we identified a specific haplotype CCACCAACT of the *DLGAP2* gene associated with schizophrenia with the odds ratio of 2.5. In addition, we also identified several rare mutations of the *DLGAP2* gene that were predicted to have pathological effect on the protein function of the *DLGAP2* gene in the patient group. Together, our data support that the *DLGAP2* is a susceptible gene of schizophrenia. Nevertheless, several rare pathological missense mutations of the *DLGAP2* gene were detected in control subjects, indicating that the clinical relevance of these putatively pathological missense mutations is not straightforward; additional environmental factors or other genetic mutations as second or third hit are needed in affected patients in order to have clinical manifestations [21], [22].

Recent studies demonstrated shared genetic etiology and neurodevelopmental pathways between different psychiatric disorder [23], [24]. We previously reported a novel chromosomal deletion of 2.4 Mb at 8p23.2-pter that contained the *DLGAP2* gene in a male patient with autistic disorder [25]. Marshall et al. also found a genomic DNA duplication intersected the *DLGAP2* gene in a patient with autism [26], and Ozgen et al. reported a classical inv dup del(8) chromosome that also included the *DLGAP2* gene in a female patient with autism [27]. In that study, we detected several common and rare genetic variants of the *DLGAP2* gene associated with autism [28]. Taken together, these studies suggest that the *DLGAP2* gene is likely a common susceptible gene between schizophrenia and autism.

Notably in the present study, we identified several patient-only variants with enhanced promoter activity, suggesting increased *DLGAP2* gene expression may contribute to the pathogenesis of schizophrenia. The *DLGAP2*-encoded SAPAP2 specifically interacts with PSD-95/SAP90 and its related proteins through the guanylate kinase domain. The protein is specifically expressed in neuronal cells, and enriched in the post-synaptic density fraction [8], [16], and plays a pivotal role in the molecular organization of synapses and in neuronal cell signaling. In the case of fragile X syndrome, which is a hereditary form of mental retardation caused by functional absence of Fragile X mental retardation protein (FMRP), Schütt *et al.* reported that mRNA of SAPAP1, 2 and 3 were part of the targets of the FMRP and increased postsynaptic SAPAP2 levels was observed in the hippocampus of FMRP knock-out mice [29], The increased SAPAP2 level may contribute to the aberrant synaptic function in patients with Fragile X syndrome. These findings provide clues to support that increased *DLGAP2* gene expression might also contribute to the pathogenesis of schizophrenia in our patients who found to have mutations with enhanced promoter activity. Nevertheless, further functional studies are needed to address the relation between increased *DLGAP2* gene and schizophrenia.

This study has several limitations. First, we lack functional study to assess the impact of missense mutations of the *DLGAP2* gene identified in this study, including one amino acid insertion in a patient, given some of them were predicted to have pathologic impact on the protein function. Second, this study was designed for case-control association analysis; hence, we did not collect family information. We need to conduct family study to trace the origin of the missense mutations indentified in this study and to assess their genotype-phenotype relationship. Third, the sample size of this study is small and the mean age of the control group is significantly higher than the patient group, hence, independent replication studies with larger sample size and better age-matched controls are needed to verify the findings from the present study.

## Materials and Methods

### Subjects

All subjects were Han Chinese from Taiwan. Patients fulfilling the diagnostic criteria for schizophrenia defined by the diagnostic and statistical manual of mental disorders-IV (DSM-IV) were recruited into this study from the Department of Psychiatry, Yuli Veterans Hospital, Hualien Armed Forced General Hospital, and Taoyuan Armed Forced General Hospital, Taiwan. The diagnosis of schizophrenia was based on clinical interview and review of medical records by senior psychiatrists with consensus. Exclusion criteria include psychosis due to general medical condition, substance-related psychosis, and mood disorder with psychotic features. Control subjects were recruited from those who received routine medical checkups from the department of family Medicine of a general hospital in eastern Taiwan. The mental status and history of mental illness of the control subjects were evaluated by a senior psychiatrist; subjects diagnosed with a DSM-IV axis I disorder were excluded. The study protocol was approved by the Ethics Committee of Yuli Veterans Hospital, Hualien Armed Forced General Hospital, Taoyuan Armed Forced General Hospital, and Tzu-Chi General Hospital, Taiwan. After giving the participants a complete description of the study, written informed consent was obtained in line with the Institutional Review Board guidelines. If the participants had a compromised ability to consent, next of kin or guardians consented on the behalf of participants after the purposes and procedures of the study were fully explained and confidentiality was ensured. The affected group comprised 523 schizophrenia patients (313 males, 210 females, mean age: 40±12 years) and the control group comprised 596 subjects (263 males, 333 females, mean age: 50±9 years). Genomic DNA was prepared from peripheral blood cells according to standard protocols.

### PCR Amplification and Direct PCR Sequencing Reaction

The human *DLGAP2* gene comprises 12 exons that span approximately 207 kb on chromosome 8p23.3. Optimal PCR primer sequences were generated to amplify each exon of the *DLGAP2* gene using Primer3 (http://frodo.wi.mit.edu/primer3/). Primer sequences, optimal annealing temperatures and size of each amplicon are listed in Table S1. Each exon of the *DLGAP2* gene was PCR amplified and subjected to direct sequencing using ABI Prism™ BigDye™ Terminator Cycle Sequencing Ready Reaction Kit Version 3.1, and an ABI autosequencer 3730 (Perkin Elmer Applied Biosystem, Foster City, CA) according to the standard protocol established in the laboratory. The authenticity of mutations identified in this study was confirmed by repeated PCR and sequencing in both directions.

### Nomenclature and Reference Sequences

The nomenclature of these sequence variations follows the “Nomenclature for description of human sequence variations” [30]. The GenBank accession numbers of the reference sequences for the genomic DNA and cDNA of the *DLGAP2* gene used in this study are NC_000008.10 and NM_004745.3, respectively.

### Genetic Association Analysis and Statistical Analysis

Differences in the allele and genotype frequencies of SNPs between schizophrenic patients and control subjects were evaluated using a χ2 test. Deviation from Hardy-Weinberg equilibrium for each SNP was checked by a chi-square goodness-of-fit test. Fisher’s exact test was used to compare whether combination of all missense mutations were significantly overrepresented in the patient group than the control group. The threshold of significance was set as p<0.05. Differences in allele, genotype, and estimated haplotype frequencies between patients and controls were evaluated using an online computer platform SHEsis (http://analysis.bio-c.cn).

### *In silico* Analysis

The putative core promoter of the *DLGAP2* gene was predicted using PROMOTER SCAN (http://www-bimas.cit.nih.gov/molbio/proscan/). The potential functional consequences of missense mutations were predicted using the Polyphen-2 (http://genetics.bwh.harvard.edu/pph2/) and the PMut (http://mmb.pcb.ub.es/PMut/) software. The alteration of putative transcription factor binding site by the promoter variant of the *DLGAP2* gene was evaluated using TESS (http://www.cbil.upenn.edu/cgi-bin/tess/tess).

### Reporter Gene Activity Assay

Genomic DNAs from the subjects were used for constructing the inserts for the reporter gene assay. For functional characterization of c.−69+9T, c.−69+13T, c.−69+47T, and c.−69+55T, sense primer (5′-tgaagatgtgcagggaatga-3′) and antisense primer (5′-gctaacgtgtgtttgtggga-3′) were used to obtain amplicon containing identified genetic variants. The PCR fragments were first cloned into pCR-II-TOPO vector (Invitrogen, CA, USA) then subcloned into the pGL3-basic vector (Promega, Madison, WI, USA) using KpnI and XhoI recognition sites, and the authenticity of each construct was verified by sequencing. SKNSH neuroblastoma cells (ATCC Number: HTB-11, ATCC, Manassas, VA, USA) were cultured on 96-well plates at 3,000 cells per well in MEM supplemented with 1 mM sodium pyruvate, 0.1 mM non-essential amino acids, penicillin-streptomycin (Invitrogen, CA, USA), and 10% fetal bovine serum. The cells were co-transfected with 200 ng of reporter plasmid and 10 ng of pRL-TK (Promega, Madison, WI, USA) as an internal control reporter using 0.5 µl of the Lipofectamine™ 2000 (Invitrogen, CA, USA) and six replicates were performed for each treatment. At 30 hours after transfection, cells were lysed and the luciferase activities were measured using the Dual-Luciferase Reporter Assay System according to the manufacturer’s instructions (Promega, Madison, WI, USA). The firefly luciferase activity was normalized against the Renilla luciferase activity in each transfection.

For functional characterization of the c.−32A, infusion primers with 15 bp extensions complementary to pGL3-basic vector ends were used to obtain amplicon containing the c.−32A (sense primer: 5′-tttctctatcgataggtacccgggtgttcaatgccgttc-3′) and antisense primer: 5′-gatcgcagatctcgagacacgtgtgcccggaacac-3′). The amplicon was cloned into pGL3-basic vector using In-Fusion HD Cloning Kit according to the manufacturer’s instruction (Clontech) and the authenticity of the clones was verified by sequencing. Transfection was performed in SKNSH neuroblastoma cell line cultured in Minimum Essential Medium (MEM) containing 5% fetal bovine serum in 24-well plates. Each well contained 10^5^ cells, 1 µg of reporter plasmid, 200 ng of pGL3-Control as an internal control reporter, and 2 µl of Lipofetamine™ 2000. Every treatment was repeated six times. At 30 hours after transfection, the luciferase activities were measured as described.

## Supporting Information

Table S1 Primer sequences, optimal annealing temperature (Ta) and size of PCR products of the DLGAP2 gene.
(DOC)
