# Supplementary material for: Role of the DLGAP2 Gene Encoding the SAP90/PSD-95-Associated Protein 2 in Schizophrenia
Source: PLoS One. 2014 Jan 8;9(1):e85373. doi: 10.1371/journal.pone.0085373 (PMC3885712; doi:10.1371/journal.pone.0085373)
Supplement: Table S1 — Primer sequences, optimal annealing temperature (Ta) and size of PCR products of the DLGAP2 gene. (DOC) [file pone.0085373.s001.doc]

# Table S1. Primer sequences, optimal annealing temperature (Ta) and size of PCR products of the *DLGAP2* gene.

| Amplicon | Forward (5’-3’) | Reverse (5’-3’) | Ta (℃) | Size (bp) |
| --- | --- | --- | --- | --- |
| Exon 1 | tgaagatgtgcagggaatga | gctaacgtgtgtttgtggga | 60 | 242 |
| Exon 2.1 | aacccacaaatctgccctct | gaagagcttctgcacggagt | 60 | 691 |
| Exon 2.2 | acgctgcagtaccagaggac | atgcagttgttatctgtcaattaaa | 60 | 697 |
| Exon 3 | tccaaaaaggagctgatgct | cgcaggcagtgggaaagt | 60 | 334 |
| Exon 4 | gtgtgggttggatggtcatt | agactggaccccagggag | 65 | 269 |
| Exon 5 | gtaacgtgatggtgaccctg | tatgcctctagagtccccgc | 65 | 326 |
| Exon 6 | agtagaccacaggctgacgg | tgctggggttacagtcagtg | 65 | 529 |
| Exon 7 | gagtggagcgtgctgagag | tgcctggcacatagaatcag | 60 | 293 |
| Exon 8 | caaaatagtcccttgcccag | aaggacaggcatgattgagg | 60 | 257 |
| Exon 9 | tcctctcagaagggctacca | cacttgaataccaaagggagga | 60 | 570 |
| Exon 10 | gctcccttggtgtgatgttt | aatgatgcccattagcttgg | 60 | 235 |
| Exon 11 | ttctccctaatccgcctctt | gggaaatttgctcgtgtgtt | 60 | 388 |
| Exon 12 | ttccacaaatcccatcctgt | gggaggaggagacagaaacc | 60 | 514 |
